# Immune cell profiles associated with human exposure to perfluorinated compounds (PFAS) suggest changes in natural killer, T helper, and T cytotoxic cell subpopulations

**DOI:** 10.1016/j.envres.2024.119221

**Published:** 2024-05-23

**Authors:** Amanda R. Tursi, Birgitte Lindeman, Anja Bråthen Kristoffersen, Hege Hjertholm, Eugenia Bronder, Monica Andreassen, Trine Husøy, Hubert Dirven, Sandra Andorf, Unni C. Nygaard

**Affiliations:** a Department of Biomedical Informatics, University of Cincinnati College of Medicine, Cincinnati, OH, USA; b Division of Biomedical Informatics, Cincinnati Children’s Hospital Medical Center, Cincinnati, OH, USA; c Norwegian Institute of Public Health, Oslo, Norway; d Department of Pediatrics, University of Cincinnati College of Medicine, Cincinnati, OH, USA; e Division of Allergy and Immunology, Cincinnati Children’s Hospital Medical Center, Cincinnati, OH, USA

**Keywords:** Mass cytometry, Immune system, Immunotoxicology, Per-and polyfluoroalkyl substances (PFAS), Perfluorooctane acid (PFOA), Perfluorooctane sulfonic acid (PFOS)

## Abstract

Per- and polyfluoroalkyl substances (PFAS) constitutes a group of highly persistent man-made substances. Recent evidence indicates that PFAS negatively impact the immune system. However, it remains unclear how different PFAS are associated with alterations in circulating leukocyte subpopulations. More detailed knowledge of such potential associations can provide better understanding into mechanisms of PFAS immunotoxicity in humans. In this exploratory study, associations of serum levels of common PFAS (perfluorooctanoic acid (PFOA), perfluorooctane sulfonic acid (PFOS), perfluorononanoic acid (PFNA), and perfluorohexane sulfonic acid (PFHxS)) and immune cell profiles of peripheral blood mononuclear cells, both with and without immunostimulation, were investigated. High-dimensional single cell analysis by mass cytometry was done on blood leukocytes from fifty participants in the Norwegian human biomonitoring EuroMix study.

Different PFAS were associated with changes in various subpopulations of natural killer (NK), T helper (Th), and cytotoxic T (Tc) cells. Broadly, PFAS concentrations were related to increased frequencies of NK cells and activated subpopulations of NK cells. Additionally, increased levels of activated T helper memory cell subpopulations point to Th2/Th17 and Treg-like skewed profiles. Finally, PFAS concentrations were associated with decreased frequencies of T cytotoxic cell subpopulations with CXCR3^+^ effector memory (EM) phenotypes. Several of these observations point to biologically plausible mechanisms that may contribute to explaining the epidemiological reports of immunosuppression by PFAS. Our results suggest that PFAS exposures even at relatively low levels are associated with changes in immune cell subpopulations, a finding which should be explored more thoroughly in a larger cohort. Additionally, causal relationships should be confirmed in experimental studies. Overall, this study demonstrates the strength of profiling by mass cytometry in revealing detailed changes in immune cells at a single cell level.

## 1. Background

Per- and polyfluoroalkyl substances (PFAS) are a diverse class of over 14,000 man-made substances characterized by hydrophobic carbon-fluoride chains that are resistant to degradation (Buck et al., 2011; Johnson et al., 2021; “PFAS|EPA: PFAS structures in DSSTox,” 2022). Numerous studies have shown environmental and human health concerns associated with PFAS production and use (Brennan et al., 2021; Johnson et al., 2021; Kotthoff et al., 2015). PFAS can bioaccumulate, several have half-lives in human serum spanning months to years, and even PFAS that have been largely ‘phased-out’ persist for years in the environment and are found in the human body (Li et al., 2018; Xu et al., 2020). Thus, there are calls for better management of PFAS and increased understanding of adverse health effects associated with human exposure (Agency for Toxic Substances and Disease Registry, 2021; Blum et al., 2015; Brennan et al., 2021; Cousins et al., 2020; EFSA Panel on Contaminants in the Food Chain (EFSA CONTAM Panel) et al., 2020; European Chemicals Agency (ECHA), 2020; “FACT SHEET: Biden-Harris Administration Launches Plan to Combat PFAS Pollution,” 2021; Kwiatkowski et al., 2020; National Toxicology Program, 2016). Recently a broad proposal to restrict around 10,000 PFAS was published by the European Chemical Agency (European Chemicals Agency (ECHA), 2023).

In recent years, there has been a particular focus on the most common PFAS and their impact on the immune system. Various animal and human studies have characterized PFAS as immunotoxicants. Notably, numerous studies report that perfluorooctanoic acid (PFOA) and perfluorooctane sulfonic acid (PFOS) exposure of mice reduced T-cell dependent IgM antibody response (TDAR) (DeWitt et al., 2008, 2009, 2016, 2009; Dong et al., 2009, 2011; Peden-Adams et al., 2008; Schrenk et al., 2020; Zheng et al., 2009). A TDAR assay is a functional assay that serves as a screening tool in animal models for detecting immunosuppression. Correspondingly, available animal studies support reduced resistance to infections in response to PFOS exposure (Guruge et al., 2008; Suo et al., 2017). Studies have also associated elevated PFAS concentrations in blood with suppressed immune responses to vaccines in both children, due to prenatal and early life exposures, and adults, particularly in pregnant women (Ait Bamai et al., 2020; Grandjean et al., 2017; Granum et al., 2013; Kaur et al., 2023; Kielsen et al., 2016; Looker et al., 2014; Porter et al., 2022; Stein et al., 2016). Both the U.S. Agency for Toxic Substances and Disease Registry and the European Food Safety Authority (EFSA) have indicated that the association between certain PFAS and a reduced vaccine response is a critical finding in humans (Agency for Toxic Substances and Disease Registry, 2021; EFSA Panel on Contaminants in the Food Chain (EFSA CONTAM Panel) et al., 2020). Additionally, the U.S. Environmental Protection Agency (EPA) recently proposed Maximum Containment Levels (MCLs) and cited evidence of an association between PFAS serum concentrations and immune outcomes (Environmental Protection Agency (EPA), 2023). In particular, the association between PFOA concentration and reduced antibody response to vaccines served as a critical effect in establishing the MCLs (Environmental Protection Agency (EPA), 2023). Reduced vaccination response is a biomarker of immunosuppression and may lead to reduced protection against infections. Some epidemiological data suggest an increased risk of childhood infections after PFAS exposure, particularly in associations with prenatal PFAS exposures (Agency for Toxic Substances and Disease Registry, 2021; EFSA Panel on Contaminants in the Food Chain (EFSA CONTAM Panel) et al., 2020; Granum et al., 2013; von Holst et al., 2021).

Although it is increasingly recognized that PFAS impact the immune system, the underlying mode of action has not yet been fully elucidated. Among the proposed hypotheses are a PFAS-driven modulation of the Th1/Th2 balance resulting in reduced B cell activation and antibody production in response to an antigen challenge, but data are not consistent across experimental models (Bell et al., 2021; Ehrlich et al., 2023; National Toxicology Program, 2016). In addition, immunomodulation mediated by dysregulation of lipid homeostasis has been proposed (Bell et al., 2021; Liang et al., 2022). Animal studies have also suggested direct suppression of B cell function and effects on the innate immune system (e.g. on natural killer cell activity) as a potential mechanism (DeWitt et al., 2016; National Toxicology Program, 2016). However, experimental support for the different hypotheses is relatively weak and requires more research. There is limited knowledge on how PFAS exposure is associated with phenotypic and functional alterations in human circulating leukocyte subclasses, including in subpopulations of T, B, and NK cells. Such information may provide further insight into mechanisms of immunotoxicity in humans and identify biomarkers of effect.

The aim of the present exploratory study was to identify (functional) immune cell profiles associated with PFAS exposure levels in humans. We analyzed associations between serum concentrations of four major PFAS (PFOS, PFOA, perfluorononanoic acid (PFNA) and perfluorohexane sulfonic acid (PFHxS)) and phenotypic and functional (after unspecific stimulation) immune cell profiles of peripheral blood mononuclear cells (PBMCs). These specific PFAS were chosen because they are generally abundant in human serum and detected in all EuroMix cohort participants. These four PFAS have been associated with negative human health effects. Their importance is emphasized by encompassing four of the six compounds in the EPA’s proposed MCLs guidelines and the four PFAS used for risk assessment in the EFSA opinion (EFSA Panel on Contaminants in the Food Chain (EFSA CONTAM Panel) et al., 2020; Environmental Protection Agency (EPA), 2023). PBMC samples from fifty participants in the Norwegian human biomonitoring EuroMix cohort with measured PFAS serum concentrations were subjected to single cell analysis by mass cytometry (CyTOF) (Husøy et al., 2019; Thépaut et al., 2021). This high-dimensional method allows for simultaneous broad and deep characterization of all the major leukocyte classes and their subsets on a single cell level. Our results illustrate how mass cytometry can reveal detailed characterization of environmental changes to immune cells and identify candidate cellular biomarkers for further epidemiological and mechanistic studies.

## 2. Methods

### 2.1. Study population

Fifty adult participants (25–70 years old) were selected from a larger (n = 144) Norwegian human biomonitoring study that is a part of the EU EuroMix project, see (Husøy et al., 2019) for additional details. The inclusion criteria were adults between the ages of 18 and 70, who were not sick within the week prior to participation. Exposure levels for a number of environmental contaminants were measured in all participants (Husøy et al., 2019; Thépaut et al., 2021). The subcohort of 50 selected for this study encompassed 27 females and 23 males, with participants spanning the PFAS exposure range. These included a set of 32 participants who were originally chosen by phthalate levels for a previously published study (Nygaard et al., 2021), where the two equally sized (n = 16 each) groups of high or low levels of phthalates were also stratified by sex (n = 8 each). To increase the range of PFAS exposure level to encompass the full exposure range of the EuroMix population, as well as to increase the sample size, an additional 18 participants were selected based on their relatively high and low levels of PFOS (n = 9 each).

### 2.2. Measurement of perfluoroalkyl and polyfluoroalkyl substances (PFAS)

Serum levels of 25 different PFAS were determined as described in Thépaut et al. (2021). Briefly, a high throughput online solid phase extraction ultra-high-performance liquid chromatography tandem mass spectrometry (UHPLC–MS/MS) method was employed. The PFAS were detected by negative electrospray ionization. Four of the PFAS (PFOS, PFOA, PFHxS and PFNA) measured in the EuroMix participants were selected for this study. Specifically, they were selected for analyses because they are abundant, extensively studied, and associated with immune suppression. The limits of quantification were 0.06 ng/ml for PFOA, 0.03 ng/ml for PFOS, 0.03 ng/ml for PFNA, and 0.012 ng/ml for PFHxS. All participants in this study had quantifiable serum levels of the four selected PFAS.

### 2.3. Immune cell profiling by mass cytometry

Immune cell profiling of unstimulated and stimulated PBMCs was performed as previously described (Nygaard et al., 2021). In short, blood samples for cytometry analysis and serum samples for PFAS determination were collected at the same time. PBMCs were isolated and frozen. After thawing 1 to 4 samples per day/batch, each sample was stained with two different metal-conjugated antibody panels. Panel 1 (Table S1) was used to broadly phenotype unstimulated cells. Panel 2 (Table S2) was used after stimulation with Phorbol 12-myristate 13-acetate (PMA) and ionomycin for 4 hours (i.e. unspecific stimulation). Brefeldin A was added to accumulate cytokines intracellularly.

Thirty-two samples were stained and acquired in batches during the fall of 2018 on a CyTOF 2 instrument (Standard BioTools/Fluidigm), as previously described (Nygaard et al., 2021). An additional 18 samples were stained and acquired one year later on a Helios instrument (Standard BioTools/Fluidigm). About 200,000 events were acquired per sample. The additional samples were added to increase statistical power and to ensure a broader spread in exposure levels for the PFAS. A Wilcoxon rank sum test was performed to compare PFAS serum levels between the set of 32 participants from the CyTOF 2 instrument with the set of 18 participants from the Helios instrument to ensure levels were not significantly different between the two sets of participants.

### 2.4. Data analyses

Due to non-normal distribution, measured PFOS, PFOA, PFNA, and PFHxS serum concentrations underwent a base-10 logarithmic transformation prior to subsequent data analysis. A plot depicting Spearman correlation coefficients between each PFAS of interest and age was made (R package psych, version 2.1.9) (Revelle, 2021). Since samples from the CyTOF 2 instrument were originally selected based on the sum of DEHP and DINP phthalate metabolite concentrations, Spearman correlation coefficients between concentrations of each PFAS and those phthalates were also calculated across all 50 samples. P-values below a cutoff of 0.05 were considered significant for both types of correlations. All mass cytometry files underwent standard preprocessing and manual gating to remove debris, doublets, and dead cells. Living CD45^+^ singlet cells were gated using single event signals for DNA content, event lengths, cisplatin, and CD45. The subsequent analysis was performed in R (version 4.0.2).

For the unstimulated samples, from each file 50,000 cells were randomly selected to ensure all samples were of the same size. The original marker intensity values in these files were transformed with a hyperbolic inverse sine (arcsinh) function with a cofactor of 5. All batches were normalized with the ‘range’ method on all markers from the Cydar R package (version 1.14.1) to correct for batch and instrument effects (Lun et al., 2017). Unsupervised clustering was done with the FlowSOM algorithm (R package FlowSOM, version 2.1.29) (Quintelier et al., 2021; Van Gassen et al., 2015). The self-organizing map was set to 100 nodes and the number of metaclusters was set to 30, with all other parameters set at the default. Clustering was done on the lineage markers specified in Table S1. Clusters that encompassed less than 1% of the total cells (12 clusters) were excluded from subsequent analysis. Linear mixed models with each PFAS as a fixed effect and instrument as the random effect were done for each cluster to extract intraclass coefficient values. The intraclass coefficients were small so it was determined the normalization process was successful. Subsequently, linear regression models were used, as the linear mixed models including instrument as the random effect were not necessary.

PFOS, PFOA, PFNA, and PFHxS levels were each individually examined as continuous variables. The proportion of cells in each cluster for each sample were compared to each PFAS by a linear regression model. Each PFAS was set as the independent variable, while the proportion of live cells (represented as values between 0 and 1) was the dependent variable. Additionally, analysis of the median expression for each of the functional markers per cluster was conducted. For each marker, clusters where more than half of samples had a median marker expression value of zero (as measured by the mass cytometer) were excluded from analysis. All other clusters that passed this threshold for an individual marker were measured via linear regression. All regression results were adjusted for multiple comparisons with a Benjamini and Hochberg false discovery rate (FDR) correction (Benjamini and Hochberg, 1995). Any adjusted P-values lower than the 10% cutoff were considered significant.

To support the results from the clustering, manual gating was done using the markers most clearly characterizing the indicated populations of interest from the unsupervised clustering. Again, linear regression was used as the statistical method. The main motivation for this manual gating was to confirm the results, as well as to see if the populations could be identified using a smaller, targeted set of markers.

For the stimulated samples data analysis, only samples that were run through the CyTOF 2 instrument (n = 32) were analyzed due to technical variability between the instruments. Manual gating was done to isolate the three broad cell populations that were identified as significant in the unstimulated samples. To ensure all samples were of equal size, cells per sample were set to fully encompass the sample with the lowest cell number. Cells were subsetted to 4,300 for NK cells, 8,000 for Tc cells, and 18,000 for Th cells. All batches were normalized with the ‘range’ method on all markers from the Cydar R package.

FlowSOM clustering was done on each of the three cell populations to isolate more specific cell clusters. The self-organizing map was set to 100 nodes for all three populations. The number of metaclusters was set at 10 for the Tc and Th populations and at 15 for the NK population. No clusters were subsequently excluded based on size. Clustering was done on all markers in panel 2 (Table S2). As was done on the unstimulated samples, PFOS, PFOA, PFNA, and PFHxS levels were each examined as continuous variables. The proportion of cells in each cluster for each sample were compared to each specific PFAS (independent variable) by a linear regression model. Subsequently, a Benjamini and Hochberg FDR correction was done and adjusted P-values lower than 10% were considered significant.

Each linear regression model that was found to be significant for a PFAS was also analyzed by linear regression with the same dependent variable and with age now the sole independent variable. The resulting P-values and Akaike information criterion (AIC) values were compared with those from the original model where the PFAS was the independent variable. This comparison was done to determine if age or the PFAS explained the outcome (i.e., cell frequency or median expression) better. When comparing AIC values, lower scores indicate a better model fit. If P-values were significant (P-value <0.1) for both a specific PFAS and age, another linear regression with that PFAS and age as the fixed independent variables was conducted. The resulting P-values for both the PFAS and age were then analyzed. Additionally, the AIC value for each multivariable model was compared with those from the corresponding univariable models.

Various visualization techniques were used to aid in examination of the results. Heatmaps depicting median expression values were created and Euclidean clustering with average linkage was used to order cell clusters (R package pheatmap, version 1.0.12) (Kolde, 2019). To create two-dimensional representations of the clusters, each sample was randomly downsampled to retain 3,000 cells to form a dataset for input into the UMAP algorithm (R package umap, version 2.7.0) (Konopka, 2020). UMAP was run with default parameters. Histogram plots showing expression distributions per marker for the cells in each cluster were made with ggplot2 (version 3.3.5) (Wickham, 2016). Linear plots depicting independent and dependent variables for each regression were also created with ggplot2. A 95% confidence interval surrounding the smoothed linear model line was included. The entire unsupervised analysis method was repeated three times to ensure robustness in the results.

## 3. Results

The study cohort consisted of 50 adult participants (25–70 years) selected from the larger EuroMix study. Table 1 illustrates participant demographics and their median serum concentrations of the selected PFAS. Since 94% of our study participants had education at a university level, we did not adjust our models for educational status due to lack of variation.

A Wilcoxon rank sum test indicated that there are no significant differences between PFOA (P-value = 0.58), PFOS (P-value = 0.88), PFNA (P-value = 0.70), PFHxS (P-value = 0.52) levels, or age (P-value = 0.86) between samples run on the two instruments. The distribution of the concentrations of the selected PFAS in the samples were found to be positively correlated (Fig. S1). Pairwise Spearman correlation coefficients ranged from 0.69 to 0.90 and all were considered significant (P-value <0.05). The selected PFAS were also positively correlated with age, albeit correlation coefficients were more moderate (Fig. S1). The pairwise Spearman correlation coefficients ranged from 0.30 to 0.56, with all considered significant (P-value <0.05). Since samples run on the CyTOF 2 instrument (n = 32) were originally selected by sum of DEHP and DiNP phthalate levels, correlations between PFAS and the individual phthalates were also examined for all samples (n = 50). None of the individual phthalates were significantly correlated with any PFAS (all P-values were above 0.05). This suggests that originally selecting some of the samples by phthalate levels did not strongly confound the current study’s results regarding PFAS.

### 3.1. NK, Th, and Tc cell subpopulations were significantly associated with PFAS in the unstimulated samples

**Fig. 1.**
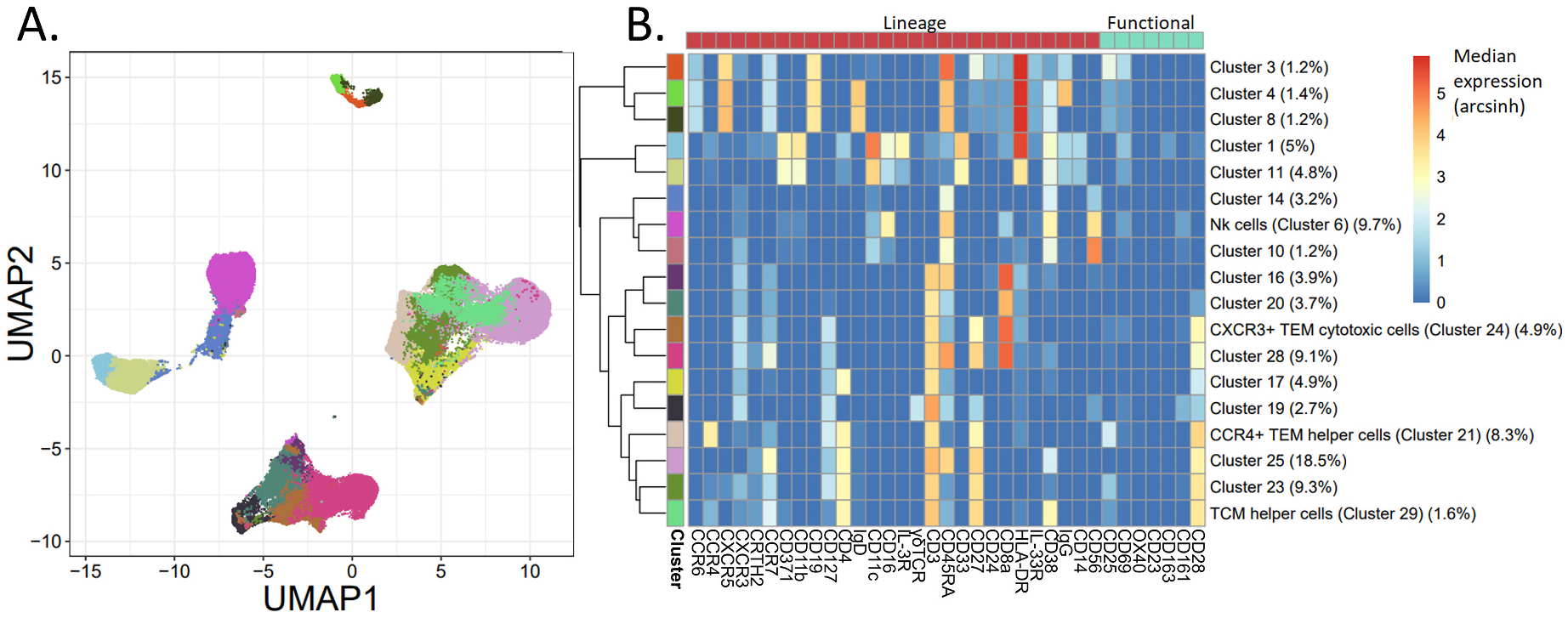
Unsupervised clustering of the unstimulated samples stained with panel 1 (n = 50). A) UMAP representation, color-mapped to the 18 FlowSOM produced clusters. The colors match to the labeled left-most side bar in Panel B (e.g. ‘dark orange’ indicates Cluster 3). B) Heatmap of the arcsinh transformed median expression values of each marker per cluster. The percent of live cells per cluster are shown in parentheses.

**Fig. 2.**
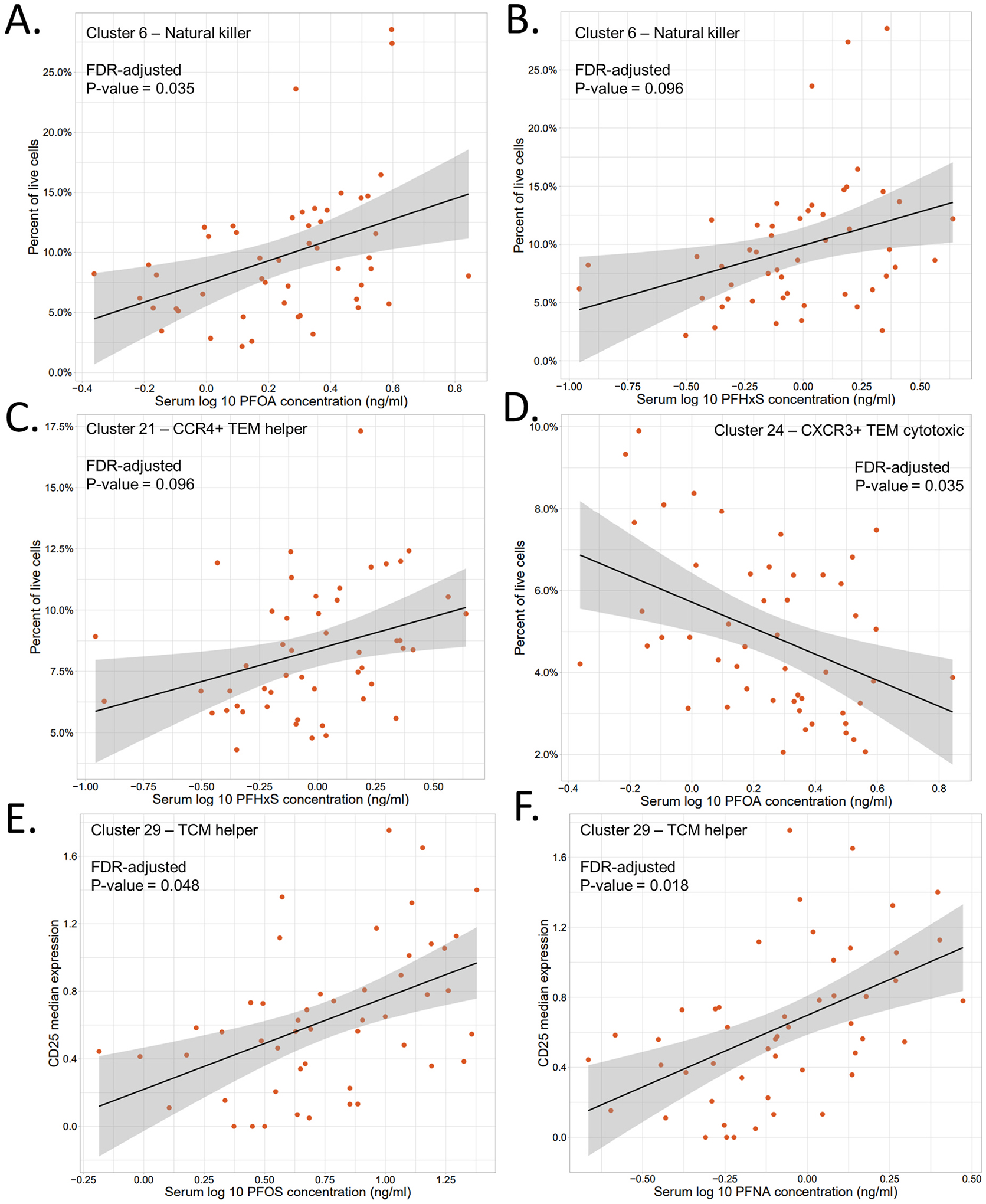
Scatterplots showing the log 10 PFAS concentration levels (ng/ml) in FlowSOM produced clusters for the 50 unstimulated samples. Linear regressions were performed to determine significance. A-D) Percent of live cells of A) a natural killer cell population (Cluster 6) versus PFOA concentrations in serum; B) a natural killer cell population (Cluster 6) versus PFHxS concentrations in serum; C) a CCR4^+^ TEM helper population (Cluster 21) versus PFHxS concentrations in serum; D) a CXCR3^+^ TEM cytotoxic population (Cluster 24) versus PFOA concentrations in serum. E-F) CD25 median expression of E) a TCM helper cell population (Cluster 29) versus PFOS concentrations in serum; F) a TCM helper cell population (Cluster 29) versus PFNA concentrations in serum.

For the unstimulated samples, FlowSOM unsupervised clustering resulted in 18 cell clusters after clusters with less than 1% of the total cells were excluded (Fig. 1). Linear regression models for PFOA, PFOS, PFNA, and PFHxS levels and cell frequencies in each of the clusters indicated that serum concentrations of PFOA and PFHxS were positively associated with a NK cell subpopulation (Cluster 6, FDR-corrected P-value = 0.035 and 0.096 respectively, Fig. 2A and B). When examining age’s possible effects on this, the linear regression model with age as the independent variable was not significant (FDR-corrected P-value = 0.34, Table 2) and the AIC value was higher than those from all four models where the individual PFAS was set as the variable (Table S3). The NK cell cluster was identified as the major NK cell population in circulation, characterized as CD56^+^, CD16^+^, CD38^+^, CD45RA^+^, CD8^low^, CD11c^low^, and CD161^low^ (Fig. 1B and S2). Manual gating was subsequently done on these markers, but its association with PFOA or PFHxS concentrations did not reach significance. However, gating on NK cells as one group (CD3^−^, CD19^−^, CD56^+^) did show a significant positive association with PFOA (P-value = 0.010, Fig. S3A, Table 2) and PFHxS (P-value = 0.039, Fig. S3B, Table 2) exposure level, as well as with PFOS and PFNA (P-value = 0.024 and 0.052, respectively). Analysis with age as the independent variable showed a possible effect from age, but overall the results indicated that PFOA and PFHxS drove the observed association for manually gated NK cells more than age did, consistent with the FlowSOM clustered result. More detailed results regarding the univariable linear regression with age and the multivariable regression with PFAS and age can be found in the supplement.

Another significant population was a T effector memory (TEM) helper cell population with a Th2 profile (Cluster 21), characterized as CD3^+^, CD4^+^, CD45RA^low^, CCR7^low^, CD27^high^, CCR4^+^, CXCR3^−^, CD127^+^, and CD25^+^ (Fig. 1B and S2). Increased concentrations of PFHxS were significantly associated with increased percentages of these CCR4^+^ TEM helper cells (FDR-corrected P-value = 0.096, Fig. 2C). The linear regression model with age as an independent variable did not result in a significant P-value in the FlowSOM cluster (but FDR-corrected P-value = 0.1, Table 2), while the AIC value was similar to that of PFHxS (AIC = −224.4 and −223.8 respectively, Table S3). Manual gating of CCR4^+^ TEM helper cells as CD3^+^, CD19^−^, CD4^+^, CD8^−^, CD45RA^low^, CCR7^low^, and CCR4^+^ also showed a significant association with PFHxS (P-value = 0.075, Fig. S3C, Table 2) but also with age (P-value = 0.058). AIC values of the univariable linear regression models and the results of the multivariable linear regression with PFHxS made it hard to disentangle the contributions of PFHxS and age on the observed association. More details can be found in the supplement.

A CXCR3^+^ TEM cytotoxic cell population (Cluster 24), characterized as CD3^+^, CD8^+^, CD45RA^low^, CCR7^low^, CD27^med^, CCR4^−^, CXCR3^+^, and CD127^+^, was found to be significantly inversely associated with PFOA levels (FDR-corrected P-value = 0.035, Fig. 1B and 2D, and S2). This was also found by manual gating of CD3^+^, CD19^−^, CD8^+^, CD4^−^, CD45RA^low^, CCR7^low^, CXCR3^+^, CD27^+^, and CD127^+^ cells (P-value = 0.082, Fig. S3D). Based on a linear regression with age as the independent variable, the variation in cell frequencies for both the FlowSOM cluster and the manually gated population could not be explained by age (Tables 2, S3 and S4).

The median expression of each functional marker was compared to the levels of the four PFAS for each of the created clusters that showed non-zero expression values for the respective marker for at least half of the samples (see methods). The only cell population that showed significance was CD25 expression within a Th population (Cluster 29), characterized as activated (CD38^+^) TCM helper cells: CD3^+^, CD4^+^, CD45RA^low^, CCR7^+^, CD27^+^, CD28^+^, CD38^+^, CD19^−^, and CD8^−^ (Fig. 1B and S2). Elevated PFOS and PFNA serum concentrations were found to be significantly associated with upregulated CD25 median expression for this TCM helper cell population (FDR-corrected P-value = 0.048 and 0.018 respectively, Fig. 2E and F). The linear regression with age as the only independent variable was not significant and the AIC was higher than those from the individual PFAS models. Increased expression of CD25 was also associated with PFOS and PFNA levels after manual gating on all Th cells as a group (CD3^+^, CD19^−^, CD4^+^, and CD8^−^) (P-value = 0.0054 and 0.030 respectively, Fig. S3E and S3F). For CD25 expression in this manually gated cell subset, the linear regression with age was also significant (P-value = 0.019). However, AIC values of the model with age as the independent variable and the results of the multivariable linear regression with each PFAS and age suggested that PFOS drove the positive association, while the respective importance of PFNA and age was less conclusive. Detailed results can be found in the supplement.

Given the positive correlation between the different PFAS, the clusters for which a significant association was identified for any of the individual PFAS were also assessed for the remaining PFAS (Table 2, S3, and S4). P-values showed that manually gated NK cells, as well as CD25 median expression in manually gated Th cells, were significantly associated with all four PFAS tested, as well as with age.

### 3.2. NK and Th cell subpopulations were significantly associated with PFAS in the stimulated samples

For the data analysis of the stimulated samples, a hypothesis-based strategy was used to target the three major cell populations (NK cells, Th cells, and Tc cells) that showed significance in the unstimulated analysis (Fig. S4). As a result, manual gating was done to isolate each of these cell types and FlowSOM clustering was done on each cell type to assess phenotypic changes, including in functional markers after the unspecific stimulation, in more detailed subpopulations. To limit the influence from technical variation (see discussion), only samples run on the CyTOF 2 instrument were included (n = 32).

**Fig. 3.**
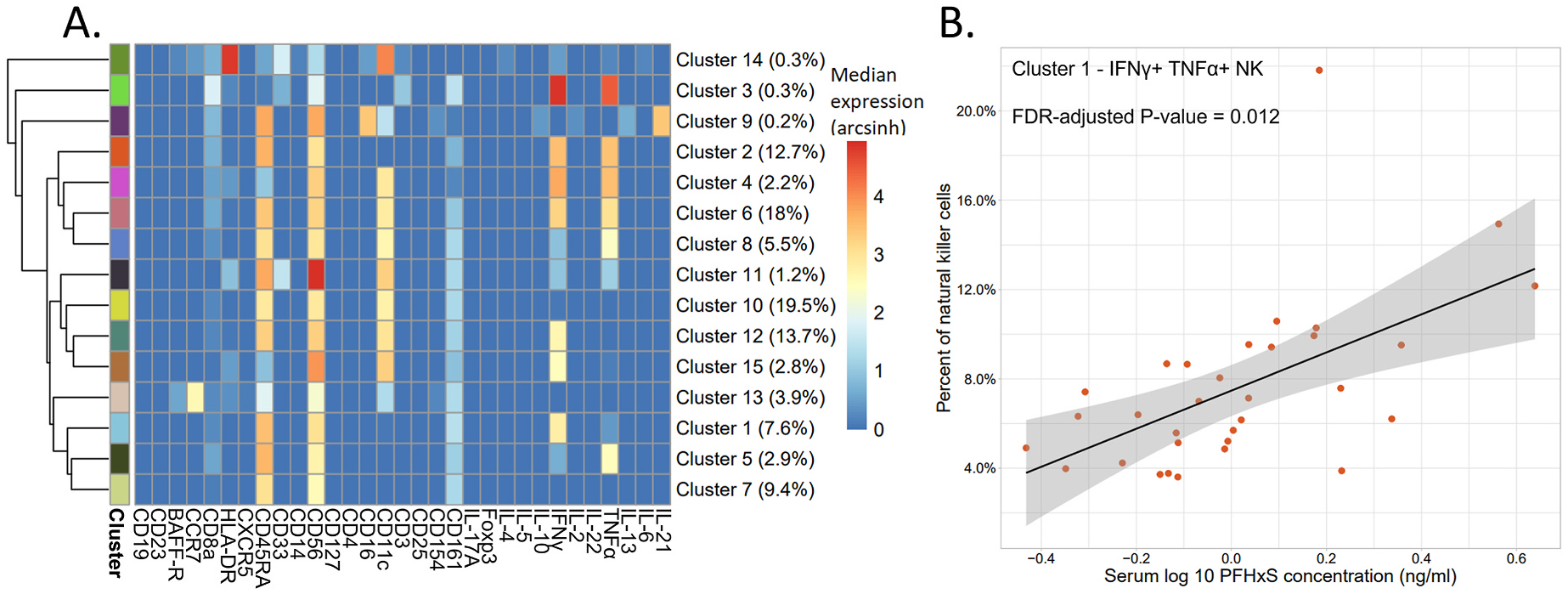
Results of the stimulated samples (n = 32) analysis on a manually gated natural killer cell population, clustered on all markers. A) Heatmap of the arcsinh transformed median expression values for each marker per cluster. The percent of natural killer cells per cluster are shown in parentheses. B) Percent of natural killer cells of an IFNγ^+^ TNFα^+^ NK population (Cluster 1) versus PFHxS concentrations in serum. Linear regressions were performed to determine significance.

For the NK cells, increased concentrations of PFHxS were associated with an increase in IFNγ^+^ TNFα^+^ NK cells (Cluster 1) characterized as CD45RA^+^, CD161^low^, CD11c^low^, IFNγ^+^, and TNFα^low^ (FDR-corrected P-value = 0.012, Fig. 3 and S5). The linear regression analysis with age as the independent variable did not reach significance and the AIC for the age model was higher than the AIC values from the PFHxS model (FDR-corrected P-value = 0.98, AIC = −114.8 and −126.4 respectively, Tables S5 and S6).

**Fig. 4.**
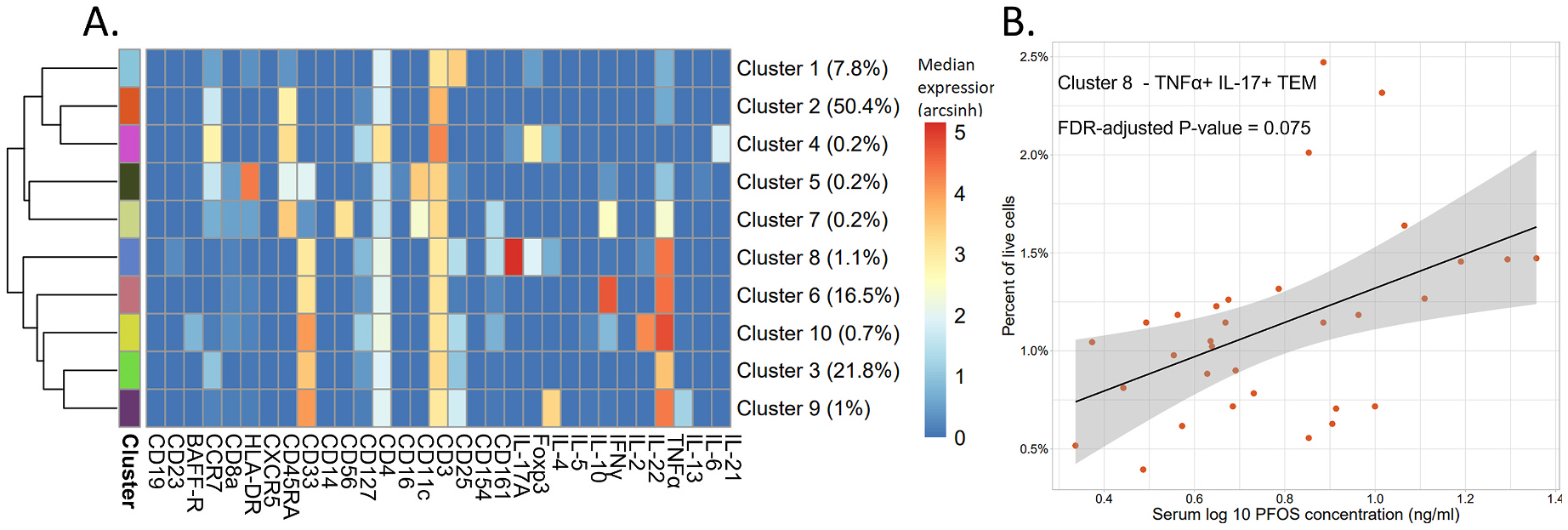
Results of the stimulated samples (n = 32) analysis on a manually gated T helper cell population. A) Heatmap of the arcsinh transformed median expression values per cluster. The percent of T helper cells per cluster are shown in parentheses. B) Percent of T helper cells of a TNFα^+^ IL-17^+^ TEM cell population (Cluster 8) versus PFOS concentrations in serum. Linear regressions were performed to determine significance.

For the T helper cells, elevated concentration of PFOS was found to be associated with higher frequency of a cluster (Cluster 8) identified as TNFα^+^ IL-17^+^ TEM cells (CD45RA^−^ CCR7^−^), the majority being Foxp3^+^ and CD25^+^, suggesting a Th17/Treg-like profile. Cells in this population were also found to be CD33^+^, CD161^+^, and IL-4^+^ (FDR-corrected P-value = 0.075, Fig. 4 and S6). When examining possible effects of age on this T helper cell population, the linear regression analysis did not reach significance and the AIC for the age model was higher than the AIC value of the univariable PFOS model (FDR-corrected P-value = 0.53, AIC = −245.7 and −252.9 respectively, Tables S5 and S6).

**Fig. 5.**
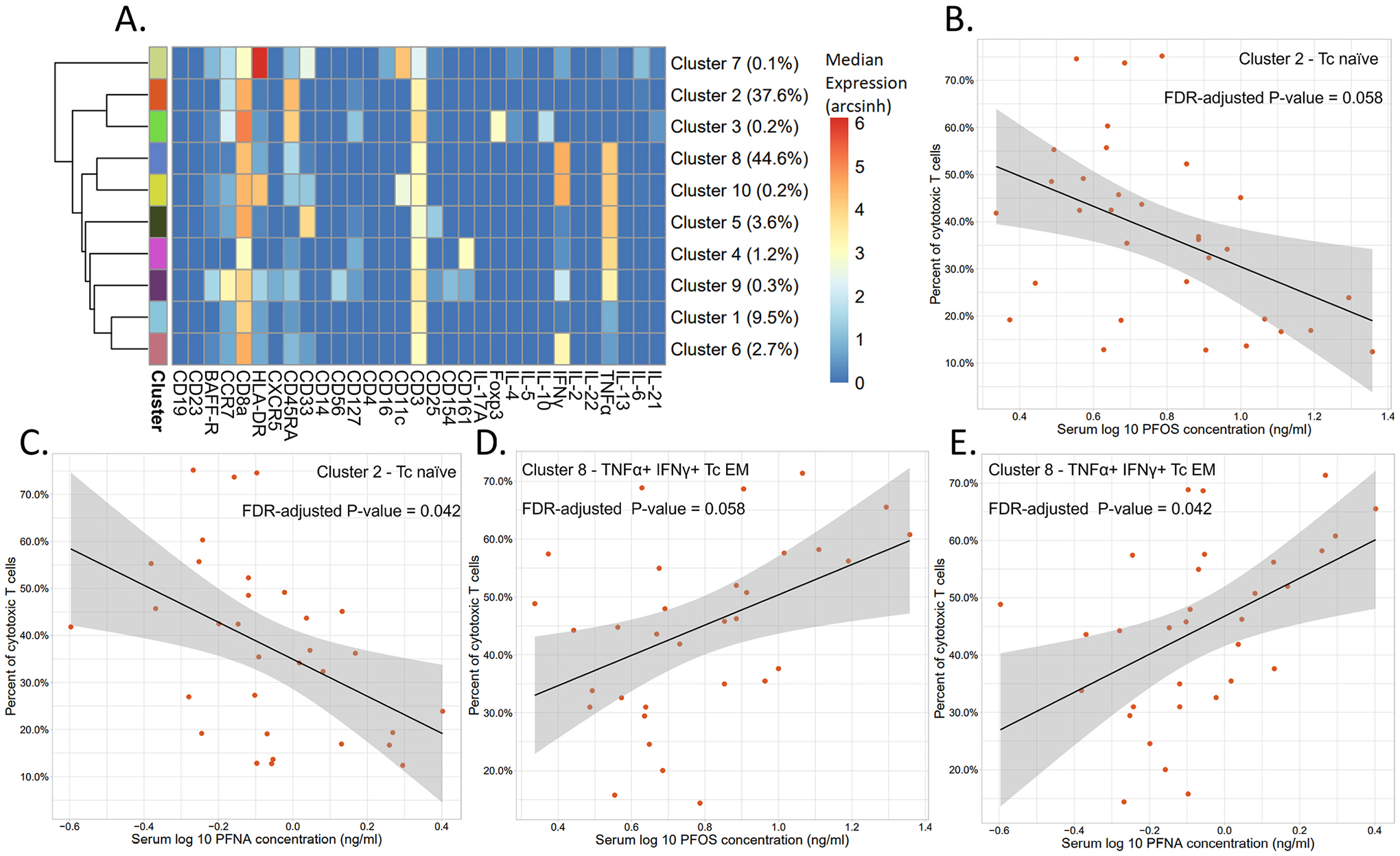
Results of the stimulated samples (n = 32) analysis on a manually gated cytotoxic T cell population, clustered by using all markers. A) Heatmap of the arcsinh transformed median expression values for each marker per cluster. The percent of cytotoxic T cells per cluster are shown in parentheses. B-E) Linear regressions were performed to determine significance. Percent of cytotoxic T cells of a Tc naïve cell population (Cluster 2) versus PFOS (B) and PFNA (C) concentrations in serum. Percent of cytotoxic T cells of a TNFα^+^ IFNγ^+^ Tc EM cell population (Cluster 8) versus PFOS (D) and PFNA (E) concentrations in serum.

Within the T cytotoxic cells, higher concentrations of PFOS and PFNA were significantly associated with decreased levels of one major cluster (Cluster 2, 37.6% of Tc) characterized as being CD45RA^high^, CCR7^+^, and CD127^low^, i.e. most probably Tc naive cells, also negative for all cytokines measured (FDR-corrected P-value = 0.058 and 0.042 respectively, Fig. 5A, 5B, 5C, and S7). Additionally, higher concentrations of PFOS and PFNA were significantly associated with increased levels of the largest cluster (Cluster 8, 44.6% of Tc) identified as a TEM (CD45RA^low^, CCR7^−^) cytotoxic cell population with a high expression of TNFα and IFNγ (FDR-corrected P-value = 0.058 and 0.042 respectively, Fig. 5A, 5D, 5E, and S7). However, when conducting a linear regression analysis with age, the FDR-corrected P-values were significant for both Cluster 2 and Cluster 8 (FDR-corrected P-value = <0.001 for both, Table S5). The AIC values for these age models were also much lower than those of the PFAS models (Table S6). A multivariable linear regression analysis with each PFAS and age revealed age to be significant in all models, while the PFAS was never significant (Table S7). Therefore, these cell subpopulations were more closely associated with age than any PFAS.

Clusters that showed significance for at least one PFAS were also assessed for the remaining PFAS in this study (Table S5), but no additional significant associations were observed. AIC scores for all these models were also calculated (Table S6).

## 4. Discussion

Despite the increasing number of studies suggesting immunomodulating effects of PFAS, few epidemiological studies have investigated PFAS associations with detailed and data-driven identification of (functional) subpopulations of leukocytes. In the present exploratory study we offer a detailed assessment of immune cell phenotypes and function (after unspecific stimulation), reporting identification of specific subpopulations within NK, Th, and Tc cells associated with exposure levels.

In the dataset generated by staining unstimulated PBMCs with the markers in panel 1, NK cell frequencies were positively associated with PFAS exposures. Specifically, both the major blood NK cell subpopulation (CD56^dim^, CD16^+^; from unsupervised clustering) and NK cells (after manual gating) were positively associated with both PFOS and PFHxS exposure levels. For manually gated NK cells, a significant association was seen for all four PFAS tested (Table 2). Our results on NKs are consistent with previous reports of a positive association between PFOS and PFHxS serum levels and absolute counts of CD16^+^ NK cells in a US study population with relatively higher PFOA exposure levels than in the present study (Lopez-Espinosa et al., 2021). The CD56 and CD16 expression levels indicate that they are mature NK cells capable of cytotoxic killing responses (Sahir et al., 2020), while CD8 expression points to a functional subtype (Ahmad et al., 2014). In the stimulated PBMCs stained with panel 2 antibodies, unsupervised clustering based on all markers also identified a NK cell subpopulation expressing IFNγ and lower levels of TNFα positively associated with PFHxS. Together, this suggests that NK cell populations may be stimulated by PFAS; both by increasing the NK cell percentage in blood and associating with a more mature (CD16) and activated (CD8, CD38, IFNγ, TNFα) phenotype. In mice studies, NK cell cytolytic activity was decreased following exposures to PFOS and PFOA (Dong et al., 2009; Jiang et al., 2020; Keil et al., 2008; Vetvicka and Vetvickova, 2013; Zheng et al., 2009). However, two studies showed a moderate increase in NK cell activity at lower internal PFAS concentrations (Dong et al., 2009; Peden-Adams et al., 2008).

In unstimulated cells, PFHxS exposure was significantly associated with increased frequency of a TEM helper cell subpopulation with chemokine receptors suggesting a Th2 profile (CCR4^+^ CXCR3^−^) (Kim et al., 2001; Yoshie and Matsushima, 2015). PFOA and PFHxS were significantly associated with decreased frequencies of a Tc TEM cell population with chemokine receptors pointing to a Th1 promoting activity (CXCR3^+^, see below). In stimulated cells, a TEM cell population with a Th17/Th2-like cytokine expression pattern of IL-17, TNFα, and IL-4, was positively associated with PFOS exposure. The majority of this population also expressed Foxp3 and CD25, suggesting a Treg-like phenotype. Finally, when examining the Th cluster 9, which clearly shows a Th2 profile (IL-4^+^, IL-13^+^, and TNFα^+^), there was close to a significant (FDR-corrected P-value = 0.12) positive trend with PFOS. Together, these observations point to a skewing towards Th2/Th17/Treg, thus away from Th1, after PFAS exposures. This would implicate suppressed Th1 responses, which may contribute to suppressed infection resistance or (cellular) vaccine responses (Aleebrahim-Dehkordi et al., 2022). Human and animal studies have shown that PFAS may alter the balance of Th1 and Th2 cytokines, but the direction of change is not consistent across studies (Abraham et al., 2020; De Guise and Levin, 2021; Dong et al., 2011; Nian et al., 2022; Zheng et al., 2011; Zhu et al., 2016). However, this variation may be explained by different study designs, like longitudinal versus cross-sectional studies, or different exposure levels, given the hypothesized non-monotonic dose response curves. Importantly, most studies assess serum bulk cytokines, which requires strong secretion (number of cells or amount per cell) to be detected and without knowledge of whether the circulating cytokines are linked to the particular cell subpopulations involved in biological effects. Single cell analysis of immune cells is therefore a useful and sensitive tool for delineating effects of PFAS on leukocyte subpopulations and their functional role.

We also observed that upregulated expression of the activation marker CD25 was associated with PFAS exposure levels within a TCM helper cell population identified in the unsupervised analyses (significant for PFOS and PFNA, for PFOS also for the manually gated Th cells). These results further suggest an association between PFAS exposure and increased activation of T helper (memory) cell subsets.

Within the T cytotoxic cell compartment, our analyses showed significant inverse associations between PFOA levels and percentage of a CXCR3^+^ TEM population (both in the unsupervised analyses and after manual gating), which was a considerable proportion (4.5%) of the cells. CXCR3 is known to be highly expressed on TEM (preferentially CD8^+^) cells and plays an important role in the trafficking of CD8^+^ T cells to peripheral sites of Th1-type inflammation (Groom and Luster, 2011). It is rapidly induced on naïve cells after activation and promotes the Th1 feedback loop including IFNγ and IFNγ-inducible CXCR3 ligands. This reduction further supports an impaired Th1-response (as discussed above). More recent studies have suggested that CXCR3 plays an important role in the contact between antigen-presenting cells and CD8^+^ T cells, affecting priming and induction of T effector and memory cells (Groom and Luster, 2011; Iwai et al., 2021; Pontes Ferreira et al., 2020). CXCR3^+^ CD8^+^ memory cells in lymph nodes engage and kill dendretic cells (DCs) as an efficient mechanism to regulate and reduce future T cell responses (Guarda et al., 2007). PFAS in experimental models impair TDAR responses (likewise, in humans they seem to impair antibody responses to vaccines), which reflects impaired responses that depend on communication between DC, T, and B cells in the lymph nodes. Therefore, our observation of reduced CXCR3^+^ TEM cells associated with PFAS exposure led us to speculate that reduced CXCR3 expression, for instance in TEM cells, can be an event in the mechanistic link, during priming and/or in the lymph node, to the reported immunosuppressive effects of PFAS.

This study has several limitations that should be considered when interpreting the findings. First, the usage of two different mass cytometers introduced instrument and batch effects that needed to be corrected. For the unstimulated samples, the suitability of the correction method used before clustering was assessed after clustering visually (Fig. S8) and by examining intraclass coefficients when running linear mixed models with instrument as a random effect. The intraclass coefficients were found to be small, indicating that the instrument effect was sufficiently addressed prior to clustering. Thus, linear regressions were used as the model. For the stimulated samples, technical variation led to the decision for a focused approach using only the samples from the CyTOF 2 instrument.

Overall, the relatively small sample size for both the unstimulated and stimulated samples should be noted. This provided a challenge in enabling more complex multiparametric analysis. Particularly, demographic factors that may affect this analysis were not included due to sample size, which should be considered when interpreting the findings. For example, sex and diet have been found to be associated with background PFAS levels in humans and could be driving some of the results presented here (Barton et al., 2020; Góralczyk et al., 2015; Haug et al., 2010; Thépaut et al., 2021). These factors should be explored more thoroughly in future studies. Socioeconomic factors that were measured indicate this subcohort has very high rates of higher education, with 94% having some level of university education. Other socioeconomic factors such as income level were not measured but based on education rates it can be inferred that these study participants were in a higher-than-average income bracket overall.

The age of the participants was considered, since it has been documented that levels of the four PFAS included in the present analyses increase with age (Haug et al., 2009; Kato et al., 2011) and PFAS concentrations were weakly but significantly correlated with age in our study. Disentangling contributions of PFAS serum levels from age is a challenge due to the positive correlation between them and the small sample size, so multiple statistical approaches were included to help interpret the results. Specifically, univariable linear regressions with age alone and multivariable linear regressions with each PFAS and age were conducted and compared to the original PFAS models. Both P-values and AIC values were examined to determine which model best fit the data and best explained the significance. In some cases, PFAS alone best explained the results, while in others unraveling how much age versus PFAS was driving results remains a challenge. However, age did seem to clearly explain two of the observations. After stimulation, PFOS and PFNA were initially found to be associated with reduced frequency of a major population of naïve Tc population without cytokines detected and increased frequency of a major subpopulation of EM Tc expressing high levels of TNFα and IFNγ. However, additional testing revealed age to be the factor primarily associated with these changes in frequency. When manually gating the populations identified by the unsupervised analyses, age appeared to contribute more to the model for 3 out of the 4 populations from the unstimulated data. In contrast, age was not found to contribute to the results from the FlowSOM analyses. We speculate that this may be due to the manually gated populations representing less specific and/or larger cell populations, which may be more impacted by the known immune senescence with age (Brodin et al., 2015; Carr et al., 2016; Simon et al., 2015; Yan et al., 2021) than by the contribution from PFAS exposure to more defined populations.

The correlation coefficient for the exposure levels for the four PFAS examined here were significant, varying between 0.69 and 0.9. This reduces the ability to identify which PFAS compounds have the strongest impact on immune responses and emphasizes the need for additional studies on other PFAS that are commonly used. In particular, emerging short-chain PFAS are increasingly used as replacements for longer chain PFAS (Ateia et al., 2019; Kaboŕe et al., 2018). Recent research suggests that increased exposure to emerging PFAS are linked to immunosuppressant effects in pregnant woman, highlighting the need for additional research focused on short-chain PFAS (Kaur et al., 2023). Studies that are composed of a larger cohort can utilize Bayesian kernel machine regression or other environmental mixture analysis methods to better understand the extent PFAS mixtures play in changes to immune subtypes (Gibson et al., 2019).

In summary, our data suggest that the different PFAS may induce changes to subpopulations of cells in blood, both within the NK, Th, and Tc subsets, in adults from a population with only background PFAS exposure levels. The overall trend was that PFAS exposure levels were associated with increased frequencies of NK cells and activated subsets of NK cells, as well as increased levels of activated T memory cell subsets, i.e., memory cell subsets with Th2 associated chemokine expression, Th2/Th17 cytokine producing TEM Th with Foxp3 expression and CD25 expression on TCMs (and T helper cells). Additionally, exposure was associated with decreased frequencies of T cytotoxic cell subsets with CXCR3^+^ EM. Several of these observations point to biologically plausible mechanisms that may contribute to explaining the epidemiological reports of immunosuppression by PFAS, like impaired Th1 responses and reduced responses in lymph nodes. Studies of these cellular biomarkers using more narrow antibody panels and flow cytometry in a larger cohort would be useful to confirm these immunological biomarkers and mechanisms hypothesized from this explorative study. Our study illustrates how high dimensional cell profiling by mass cytometry can reveal mechanistic and biomarker clues of environmental changes to immune cells. The strength of this method is identification of a wide range of markers on a single cell level, allowing for detailed characterization of effects on smaller cell subsets.

## Supplementary Material

Supplementary 1a

## Figures and Tables

**Table 1:** Participant demographics and median concentrations of the selected PFAS; combined as well as stratified by the instrument that was used for acquisition. IQR: interquartile range.

Characteristic	CyTOF 2	Helios	Combined
Number of participants	32	18	50
Sex; n female (%)	16 (50%)	11 (61%)	27 (54%)
Age (years); median (IQR)	40.5 (33.5, 52)	42.5 (33, 49.5)	41 (32.5, 51.75)
PFOA (ng/ml); median (IQR)	1.99 (1.46, 2.50)	1.51 (0.992, 3.13)	1.96 (1.23, 2.96)
PFOS (ng/ml); median (IQR)	5.15 (4.12, 8.44)	7.73 (1.76, 15.4)	5.15 (3.25, 11.9)
PFNA (ng/ml); median (IQR)	0.805 (0.618, 1.14)	0.783 (0.401, 1.39)	0.805 (0.545, 1.36)
PFHxS (ng/ml); median (IQR)	0.976 (0.736, 1.50)	0.725 (0.409, 2.14)	0.957 (0.612, 1.57)

**Table 2:** Significant results of the unstimulated samples (n = 50) analysis. Cell subpopulations that showed significance (FDR-corrected P-value <0.1) in at least one PFAS when clustered with FlowSOM that were also confirmed by manual gating are depicted. For these cell subpopulations, the resulting FDR-corrected P-value and regression coefficients from linear regression with age as the independent variable is also shown. Regression coefficients and FDR-corrected P-values (<0.1 in bold) are shown for the FlowSOM clusters and uncorrected P-values are shown for the manually gated populations. P-values that were determined to be significant in both the unsupervised (FDR-corrected P-value <0.1) and manually gated (P-value <0.1) analysis are italicized.

Cell Population	PFOA	PFOS	PFNA	PFHxS	Age
**FlowSOM, FDR-corrected P-value (Regression Coefficient)**					
NK cells (Cluster 6)	***0.035 (0.086)***	0.18 (0.054)	0.15 (0.064)	***0.096 (0.058)***	0.34 (0.0013)
CCR4^+^ TEM helper cells (Cluster 21)	0.10 (0.034)	0.35 (0.018)	0.15 (0.030)	***0.096 (0.027)***	0.10 (0.00076)
CXCR3^+^ TEM cytotoxic cells (Cluster 24)	***0.035 (−0.032)***	0.35 (−0.015)	0.15 (−0.021)	**0.096 (−0.020)**	0.97 (−9.2e-6)
Median expression of CD25, TCM cells (Cluster 29)	0.20 (0.68)	***0.048 (0.54)***	***0.018 (0.81)***	0.16 (0.55)	0.24 (0.011)
**Manual Gating, P-value (Regression Coefficient)**					
NK cells (CD3^−^, CD19^−^, CD56^+^)	***0.010 (0.080)***	**0.024 (0.050)**	**0.052 (0.061)**	***0.039 (0.052)***	**0.057 (0.0013)**
CCR4^+^ TEM helper cells (CD3^+^, CD19^−^ CD4^+^, CD8^−^ CD45RA^low^, CCR7^low^, CCR4^+^)	0.14 (0.023)	0.39 (0.0092)	0.35 (0.014)	***0.075 (0.021)***	**0.058 (0.00063)**
CXCR3^+^ TEM cytotoxic cells (CD3^+^, CD19^−^ CD8^+^, CD4^−^ CD45RA^low^, CCR7^low^, CXCR3^+^, CD27^+^, CD127^+^)	***0.082 (−0.0034)***	0.26 (−0.0015)	0.29 (−0.0021)	0.36 (−0.0014)	0.71 (1.6e-5)
Median expression of CD25, Th cells (CD3^+^, CD19^−^, CD4^+^, CD8^−^)	**0.0046 (4.76)**	***0.0054 (3.26)***	***0.030 (3.64)***	**0.013 (3.33)**	**0.019 (0.086)**

## Data Availability

The authors do not have permission to share data.
