# Supplementary material for: Immune cell profiles associated with human exposure to perfluorinated compounds (PFAS) suggest changes in natural killer, T helper, and T cytotoxic cell subpopulations
Source: Environ Res. Author manuscript; Available in PMC 2025 Mar 25. (PMC11934339; doi:10.1016/j.envres.2024.119221)
Supplement: Supplementary 1a [file NIHMS2066274-supplement-Supplementary_1a.docx]

**Supplemental results**

**Manually gated NK cells (CD3-, CD19-, CD56+)**

The possible effects of age were examined on the NK cells through both univariable and multivariable linear regression models. The linear regression model with age as the independent variable was significant (P-value = 0.057), although the AIC value was still higher than those from the PFOA and PFHxS models (AIC = -140.4, -143.5, and -141.1 respectively, Table S3). A multivariable linear regression was done to help resolve the effect age may be having compared to those of PFOA and PFHxS on the model. For these manually gated NK cells, age did not reach significance while PFOA reached significance and PFHxS was near significance (P-value = 0.038 and 0.10 respectively, Table S4). The AIC values of both the PFOA and age model (AIC = -143.1) and the PFHxS and age model (AIC = -141.3) were respectively very similar to that of PFOA (AIC = −143.5) and PFHxS (AIC = -141.1) alone, indicating similar fits.

**Manually gated CCR4+ TEM helper cells (CD3+, CD19-, CD4+, CD8-, CD45RAlow, CCR7low, CCR4+)**

The AIC value of the linear regression with age as the independent variable was similar to that of the model with PFHxS as the independent variable (AIC = -214.0 and -213.6 respectively, Table S3). The multivariable linear regression with PFHxS and age resulted in similar respective P-values, with none reaching significance (P-value = 0.18 and 0.14 respectively, Table S4). The AIC of this multivariable model was -214.0.

**Median expression of CD25, manually gated Th cells (CD3+, CD19-, CD4+, CD8-)**

The AIC of the univariable linear regression age model was higher than that of the PFOS model (AIC = 256.5 and 254.1 respectively) but slightly lower than that of the PFNA model (AIC = 257.3, Table S3). The multivariable linear regression with PFOS and age was significant for PFOS but not age (P-value = 0.047 and 0.19 respectively), with an AIC value of 254.2 (Table S4). The model with PFNA and age was not significant for PFNA or age (P-value = 0.27 and 0.16 respectively). The AIC value for this model was 257.2.

Table S1. Mass cytometry antibody panel 1, used for the unstimulated samples. * indicates lineage markers that were used for clustering with the FlowSOM algorithm.

| **Target** | **Label** | **Clone** | **Provider** |
| --- | --- | --- | --- |
| *CCR6/CD196 | 141Pr | 11A9 | Fluidigm |
| *CCR4/CD194 | 149Sm | 205410 | Fluidigm |
| *CXCR5/CD185 | 153Eu | RF8B2 | Fluidigm |
| *CXCR3/CD183 | 156Gd | G025H7 | Fluidigm |
| *CRTH2/CD294 | 163Dy | BM16 | Fluidigm |
| *CCR7/CD197 | 167Er | G043H7 | Fluidigm |
| *CD371 | 173Yb | 50C1 | Fluidigm |
| *CD11b | 209Bi | ICRF44 | Fluidigm |
| *CD19 | 142Nd | HIB19 | Fluidigm |
| * IL-7Ra/CD127 | 143Nd | A019D5 | Fluidigm |
| *CD4 | 145Nd | RPA-T4 | Fluidigm |
| *IgD | 146Nd | TA6-2 | Fluidigm |
| *CD11c | 147Sm | Bu15 | Fluidigm |
| *CD16 | 148Nd | 3G8 | Fluidigm |
| *IL-3R/CD123 | 151Eu | 6H6 | Fluidigm |
| * γδTCR | 152Sm | 11F2 | Fluidigm |
| *CD3 | 154Sm | UCHT1 | Fluidigm |
| *CD45RA | 155Gd | HI100 | Fluidigm |
| *CD33 | 158Gd | WM53 | Fluidigm |
| *CD27 | 162Dy | L128 | Fluidigm |
| *CD24 | 166Er | ML5 | Fluidigm |
| *CD8a | 168Er | SK1 | Fluidigm |
| *HLA-DR | 170Er | L243 | Fluidigm |
| *IL-33R | 171Yb | Sf21 | R&D systems |
| *CD38 | 172Yb | H1T2 | Fluidigm |
| *IgG | 174Yb | MNK-49 | BioLegend |
| *CD14 | 175Lu | M5E2 | Fluidigm |
| *CD56 | 176Yb | NCAM16.2 | Fluidigm |
| IL-2R/CD25 | 169Tm | 2A3 | Fluidigm |
| CD69 | 144Nd | FN90 | Fluidigm |
| OX40/CD134 | 150Nd | ACT35 | Fluidigm |
| CD23 | 164Dy | EBVCS-5 | Fluidigm |
| CD163 | 165Ho | GHI/61 | Fluidigm |
| CD161 | 159Tb | HP-3G10 | Fluidigm |
| CD28 | 160Gd | CD28.2 | Fluidigm |

Table S2. Mass cytometry antibody panel 2, used for the stimulated samples. All markers were used for clustering with the FlowSOM algorithm.

| **Target** | **Label** | **Clone** | **Provider** |
| --- | --- | --- | --- |
| CD19 | 142Nd | HIB19 | Fluidigm |
| CD23 | 164Dy | EBVCS-5 | Fluidigm |
| BAFF-R/CD268 | 166Er |  | BioLegend |
| CCR7/CD197 | 167Er | G043H7 | Fluidigm |
| CD8a | 168Er | SK1 | Fluidigm |
| HLA-DR | 170Er | L243 | Fluidigm |
| CXCR5/CD185 | 153Eu | RF8B2 | Fluidigm |
| CD45RA | 155Gd | HI100 | Fluidigm |
| CD33 | 158Gd | WM53 | Fluidigm |
| CD14 | 175Lu | M5E2 | Fluidigm |
| CD56 | 176Yb | NCAM16.2 | Fluidigm |
| IL-7Ra/CD127 | 143Nd | AD19D5 | Fluidigm |
| CD4 | 145Nd | RPA-T4 | Fluidigm |
| CD16 | 148Nd | 3G8 | Fluidigm |
| CD11c | 147Sm | Bu15 | Fluidigm |
| CD3 | 154Sm | UCHT1 | Fluidigm |
| IL-2R/CD25 | 169Tm | 2A3 | Fluidigm |
| CD45 | 89Y | HI30 | Fluidigm |
| CD154/CD40L | 173Yb |  | BioLegend |
| CD161 | 174Yb |  | BioLegend |
| IL-17A | 161Dy | BL168 | Fluidigm |
| Foxp3 | 162Dy | 259D/C7 | Fluidigm |
| IL-4 | 163Dy | MP4-25D2 | Fluidigm |
| IL-5 | 151Eu | TRFK5 | Fluidigm |
| IL-10 | 156Gd | JES-9D7 | BioLegend |
| IFNγ | 165Ho | B27 | Fluidigm |
| IL-2 | 144Nd | MQ1-17H12 | Fluidigm |
| IL-22 | 150Nd | 22URTI | Fluidigm |
| TNFα | 152Sm | Mab11 | Fluidigm |
| IL-13 | 159Tb | JES10-5A2 | BioLegend |
| IL-6 | 171Yb | MQ2-BA5 | BioLegend |
| IL-21 | 172Yb | 3A3-N2 | Fluidigm |

**Table S3. Akaike information criterion (AIC) values that correspond to the clusters that had a least one** significant result for the linear regression analysis in the unstimulated samples (n=50). AIC values for the univariable linear regressions for each PFAS and age are shown.

| **Cell Population** | **PFOA** | **PFOS** | **PFNA** | **PFHxS** | **Age** |
| --- | --- | --- | --- | --- | --- |
| ***FlowSOM*** | | | | | |
| NK cells (Cluster 6) | −149.3 | −147.5 | −145.3 | −146.7 | −144.4 |
| CCR4+ TEM helper cells (Cluster 21) | −223.7 | −221.0 | −222.7 | −223.8 | −224.4 |
| CXCR3+ TEM cytotoxic cells (Cluster 24) | −255.4 | −250.0 | −249.9 | −251.7 | −245.6 |
| Median expression of CD25, TCM cells (Cluster 29) | 58.5 | 55.7 | 53.8 | 58.1 | 62.1 |
| ***Manual Gating*** | | | | | |
| NK cells (CD3-, CD19-, CD56+) | -143.5 | -142.0 | -140.6 | -141.1 | -140.4 |
| CCR4+ TEM helper cells (CD3+, CD19-, CD4+, CD8-, CD45RAlow, CCR7low, CCR4+) | -212.6 | -211.0 | -211.1 | -213.6 | -214.0 |
| CXCR3+ TEM cytotoxic cells (CD3+, CD19-, CD8+, CD4-, CD45RAlow, CCR7low, CXCR3+, CD27+, CD127+) | -419.5 | -417.6 | -417.5 | -417.2 | -416.4 |
| Median expression of CD25, Th cells (CD3+, CD19-, CD4+, CD8-) | 253.8 | 254.1 | 257.3 | 255.7 | 256.5 |

**Table S4. Cell subpopulations from unstimulated samples that had a significant P-value (<0.1) from the univariable linear regression model with at least one PFAS and with age are shown. Multivariable linear regressions with each PFAS and age used as independent variables were performed. The dependent variable was the proportion of manually gated NK cells and CCR4+ TEM helper cells and the median expression of CD25 for the manually gated Th cells. Each model’s P-value, regression coefficient, and Akaike information criterion (AIC) values are shown. P-values that were significant are highlighted in red and regression coefficient values are in the parentheses.**

| **Cell population** | **PFOA + age** | | | | **PFOS + age** | | | | **PFNA + age** | | | | **PFHxS + age** | | |
| --- | --- | --- | --- | --- | --- | --- | --- | --- | --- | --- | --- | --- | --- | --- | --- |
|  | **PFOA**  **P-value** | **Age**  **P-value** | **AIC** | **PFOS**  **P-value** | | **Age**  **P-value** | **AIC** | **PFNA**  **P-value** | | **Age**  **P-value** | **AIC** | **PFHxS**  **P-value** | | **Age**  **P-value** | **AIC** |
| NK cells | 0.038 (0.068) | 0.23 (0.00083) | -143.1 | 0.11 (0.039) | | 0.29 (0.00078) | -141.2 | 0.28 (0.040) | | 0.30 (0.00083) | -139.7 | 0.10 (0.042) | | 0.15 (0.00099) | -141.3 |
| CCR4+ TEM helper cells | 0.35  (0.015) | 0.13 (0.00052) | -213.0 | 0.97 (0.00039) | | 0.093 (0.00062) | -212.0 | 0.91  (-0.0019) | | 0.10 (0.00065) | -212.0 | 0.18 (0.016) | | 0.14 (0.00050) | -214.0 |
| Median expression of CD25, Th cells | 0.025 (3.87) | 0.11 (0.059) | 253.1 | 0.047 (2.53) | | 0.19 (0.052) | 254.2 | 0.27 (2.15) | | 0.16 (0.060) | 257.2 | 0.048 (2.66) | | 0.073 (0.066) | 254.3 |

Table S5. Clusters that showed significance (FDR-corrected P-value < 0.1) in at least one PFAS when clustered with FlowSOM on manually gated populations of the stimulated samples (n=32) from the CyTOF2 instrument. FDR-corrected P-values and regression coefficients are shown and significant ones are highlighted in red.

| **Cell Population** | **PFOA** | **PFOS** | **PFNA** | **PFHxS** | **Age** |
| --- | --- | --- | --- | --- | --- |
| ***NK cells, FDR-corrected P-value (Regression Coefficient)*** | | | | | |
| Cluster 1 | 0.57 (0.058) | 0.29 (0.053) | 0.97 (0.031) | 0.012 (0.085) | 0.98 (4e-04) |
| ***Tc cells, FDR-corrected P-value (Regression Coefficient)*** | | | | | |
| Cluster 2 | 0.75 (-0.14) | 0.058 (-0.32) | 0.042 (-0.39) | 0.94 (-0.037) | <0.001 (-0.012) |
| Cluster 8 | 0.90 (0.032) | 0.058 (0.26) | 0.042 (0.33) | 0.94 (-0.014) | <0.001 (0.0093) |
| ***Th cells, FDR-corrected P-value (Regression Coefficient)*** | | | | | |
| Cluster 8 | 0.85 (0.0022) | 0.075 (0.0087) | 0.27 (0.0069) | 0.39 (0.0049) | 0.53 (1e-4) |

Table S6. **Akaike information criterion (AIC) values that correspond to the FlowSOM clusters that had a least one** significant result for the linear regression analysis of the stimulated samples (n=32) from the CyTOF2 instrument.

| **Cell Population** | **PFOA** | **PFOS** | **PFNA** | **PFHxS** | **Age** |
| --- | --- | --- | --- | --- | --- |
| ***NK cells*** | | | | | |
| Cluster 1 | -118.1 | -118.9 | -115.3 | -126.4 | −114.8 |
| ***Tc cells*** | | | | | |
| Cluster 2 | -12.8 | -19.0 | −19.451 | −12.0 | −37.1 |
| Cluster 8 | -24.2 | -31.0 | −32.010 | −24.1 | −46.6 |
| ***Th cells*** | | | | | |
| Cluster 8 | −245.4 | −252.9 | -248.2 | −247.1 | −245.7 |

Table S7. **Cell subpopulations from stimulated samples that had a significant** **FDR-corrected P-value (<0.1) from the univariable linear regression model with at least one PFAS and with age are shown. Multivariable linear regressions with each PFAS and age used as independent variables were performed. The dependent variable was the proportion of live cells for both clusters. Each model’s P-value, regression coefficient, and Akaike information criterion (AIC) values are shown. FDR-corrected P-values that were significant are highlighted in red and regression coefficient values are in the parentheses.**

| **Cell population** | **PFOA + age** | | | **PFOS + age** | | | **PFNA + age** | | | **PFHxS + age** | | |
| --- | --- | --- | --- | --- | --- | --- | --- | --- | --- | --- | --- | --- |
|  | **PFOA**  **P-value** | **Age**  **P-value** | **AIC** | **PFOS**  **P-value** | **Age**  **P-value** | **AIC** | **PFNA**  **P-value** | **Age**  **P-value** | **AIC** | **PFHxS**  **P-value** | **Age**  **P-value** | **AIC** |
| Tc cells (Cluster 2) | 0.86 (0.024) | **<0.001**  **(-0.012)** | −35.1 | **0.87**  **(-0.063)** | **<0.001**  **(-0.011)** | −35.5 | 0.99  (0.040) | <0.001 (-0.011) | −35.2 | 0.93  (-0.039) | **<0.001 (-0.012)** | −35.3 |
| Tc cells (Cluster 8) | **0.79**  **(-0.10)** | **<0.001 (0.0098)** | −46.0 | **0.87 (0.059)** | **<0.001 (0.0086)** | −45.1 | 0.99 (0.060) | 0.0013 (0.0087) | −44.9 | 0.93  (-0.012) | **<0.001 (0.0093)** | −44.7 |


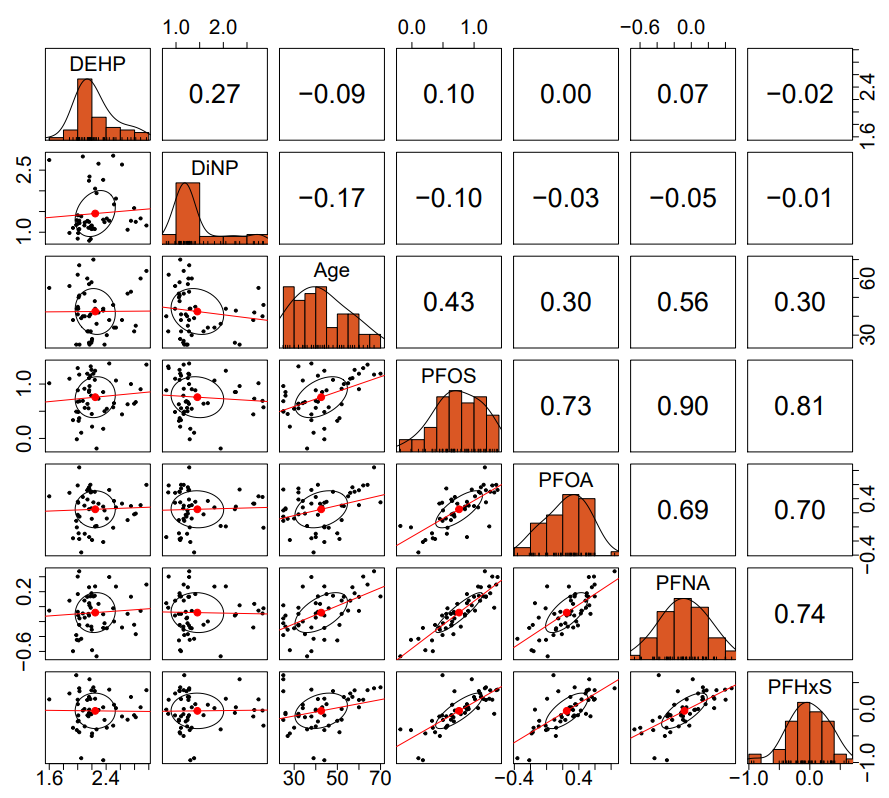


Figure S1. Plot displaying correlations between log 10 concentrations (ng/ml) of each of the four selected PFAS, age (years), DEHP (log molecular weight adjusted sum of metabolites), and DiNP (log molecular weight adjusted sum of metabolites). The middle horizontal displays histograms for each PFAS, age, and phthalates. The lower left scatter plots include linear regression lines and correlation ellipses. The upper right squares depict the Spearman correlation coefficients.


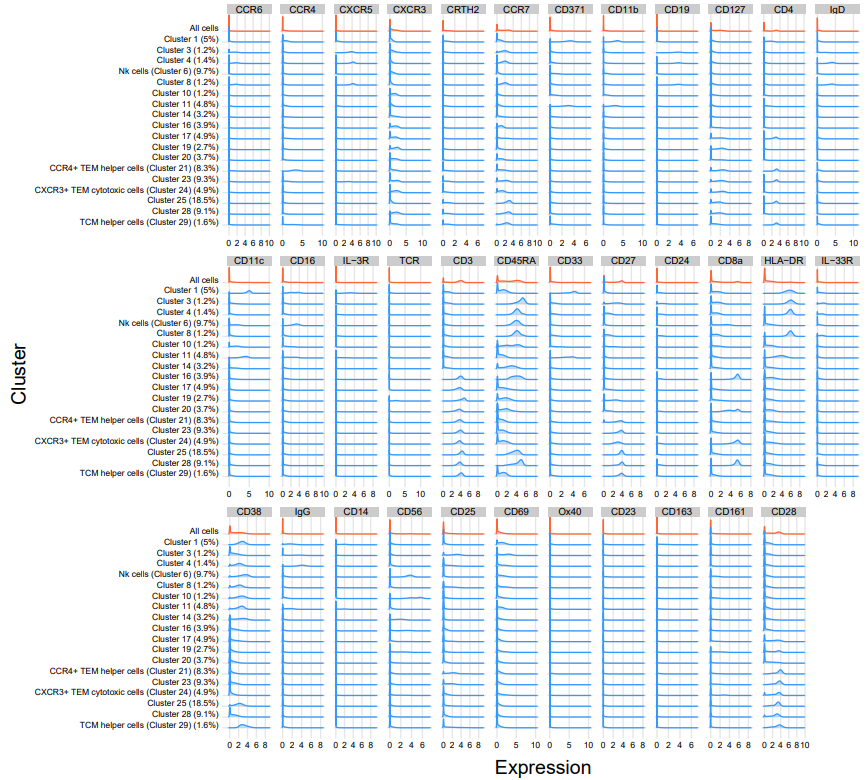


Figure S2. Histogram plots showing the expression distribution (arcsinh transformed) per cluster from the unstimulated samples. The percent of live cells per cluster are shown in parentheses.


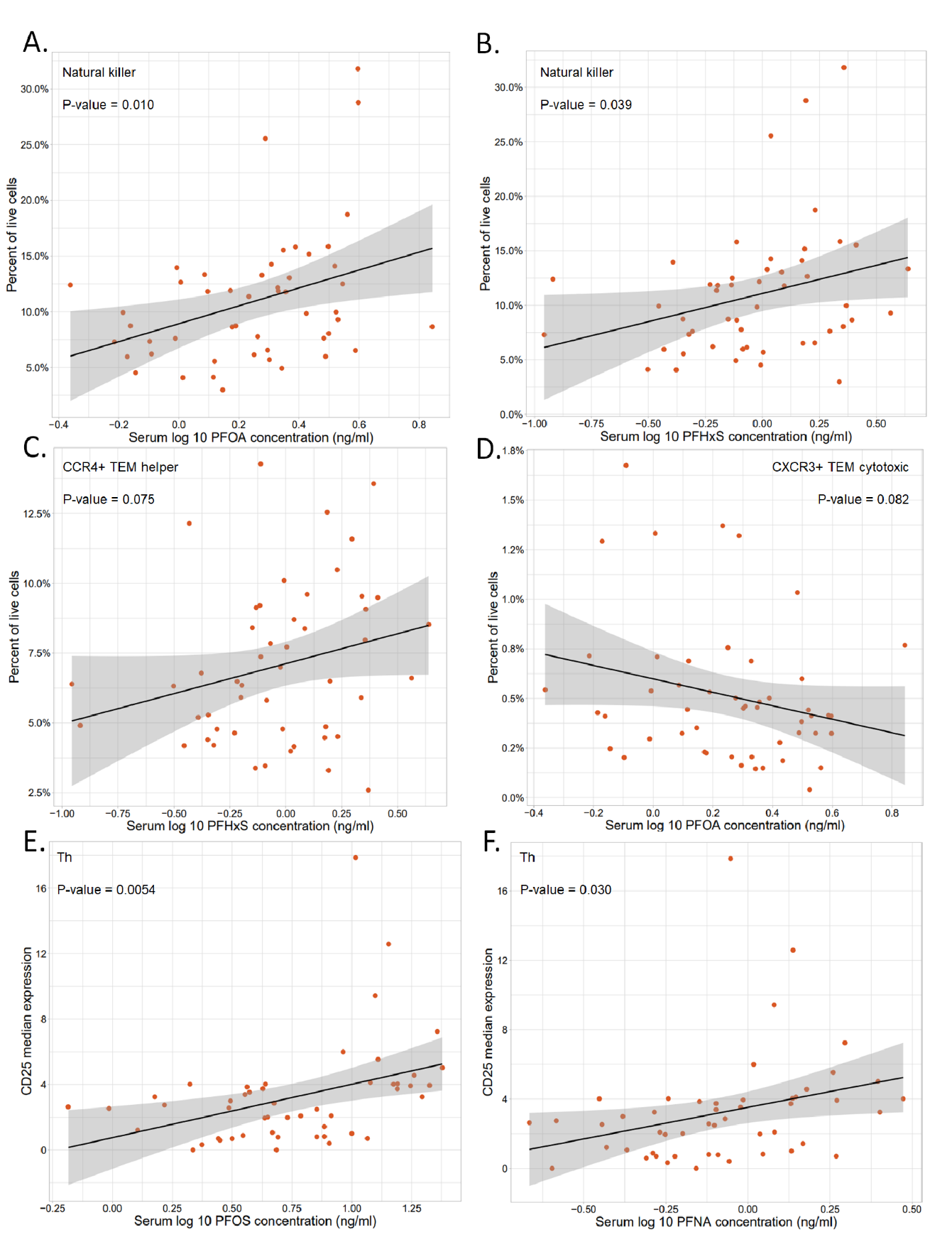


Figure S3. Scatterplots showing the log 10 PFAS concentration levels (ng/ml) in manually gated populations for the 50 unstimulated samples. Linear regressions were performed to determine significance. A-D) Percent of live cells of A) natural killer cells (CD3-, CD19-, CD56+) versus PFOA concentrations in serum; B) natural killer (CD3-, CD19-, CD56+) cells versus PFHxS concentrations in serum; C) a CCR4+ TEM helper cell population (CD3+, CD19-, CD4+, CD8-, CD45RAlow, CCR7low, CCR4+) versus PFHxS concentrations in serum; D) a CXCR3+ TEM cytotoxic cell population (CD3+, CD19-, CD8+, CD4-, CD45RAlow, CCR7low, CXCR3+, CD27+, CD127+) versus PFOA concentrations in serum. E-F) CD25 median expression of the Th cell population (CD3+, CD19-, CD4+, CD8-) versus PFOS (E) and PFNA (F) concentrations in serum.


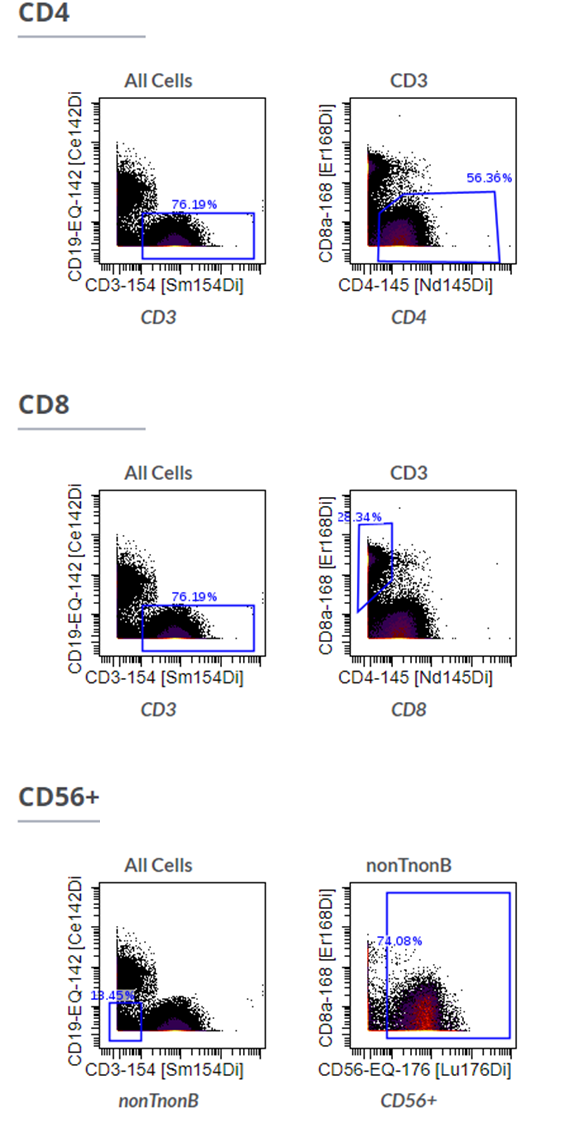


Figure S4. Manual gating strategies for isolating subpopulations in the stimulated analyses. Three different subpopulations (depicted from top to bottom, respectively) of Th cells (CD3+, CD19-, CD4+), Tc cells (CD3+, CD19-, CD8+), and NK cells (CD3-, CD19-, CD56+) were isolated prior to unsupervised analyses.


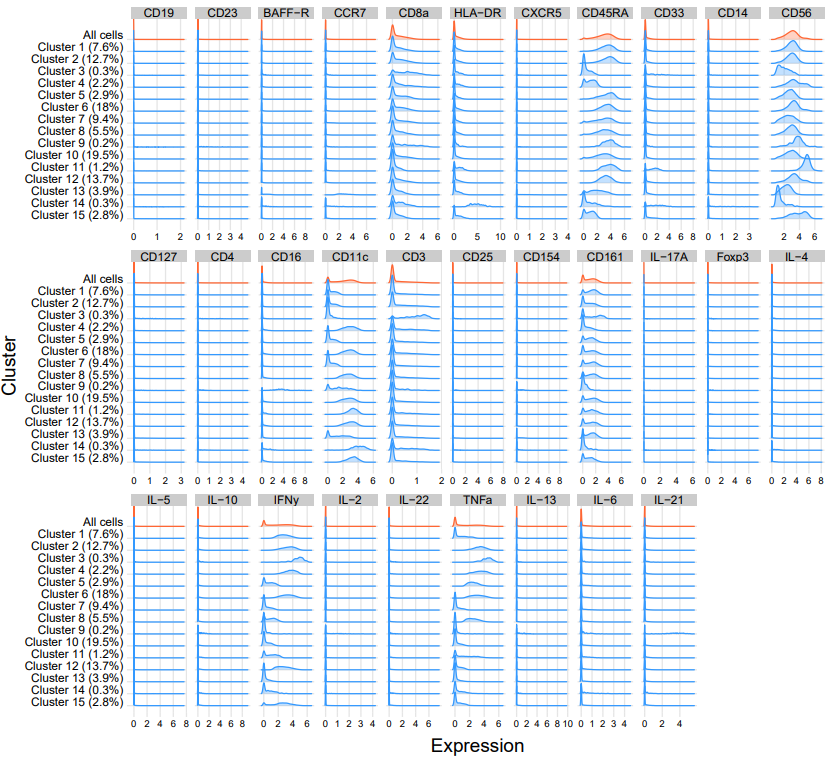


Figure S5. Histogram plots showing the expression distribution (arcsinh transformed) per cluster in natural killer cells (CD3- CD19- CD56+) from the stimulated samples.


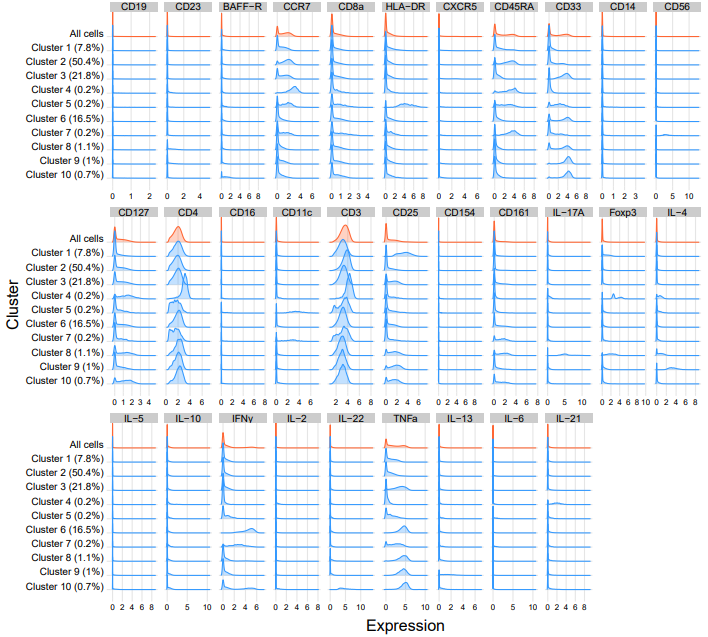


Figure S6. Histogram plots showing the expression distribution (arcsinh transformed) per cluster in Th cells (CD3+, CD19-, CD4+) from the stimulated samples.


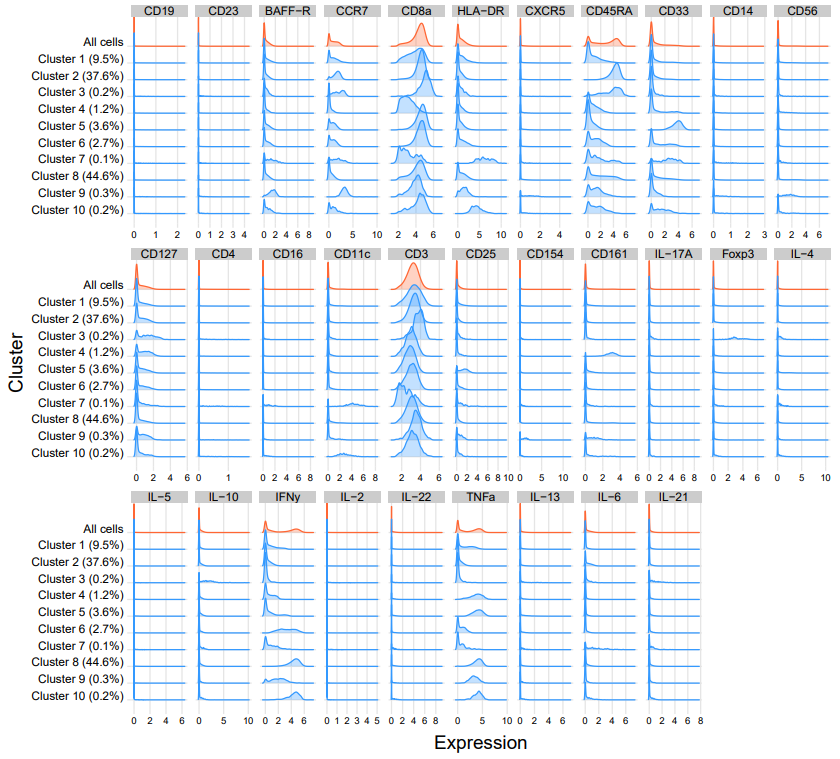


Figure S7. Histogram plots showing the expression distribution (arcsinh transformed) per cluster in Tc cells (CD3+, CD19-, CD8+), from the stimulated samples.


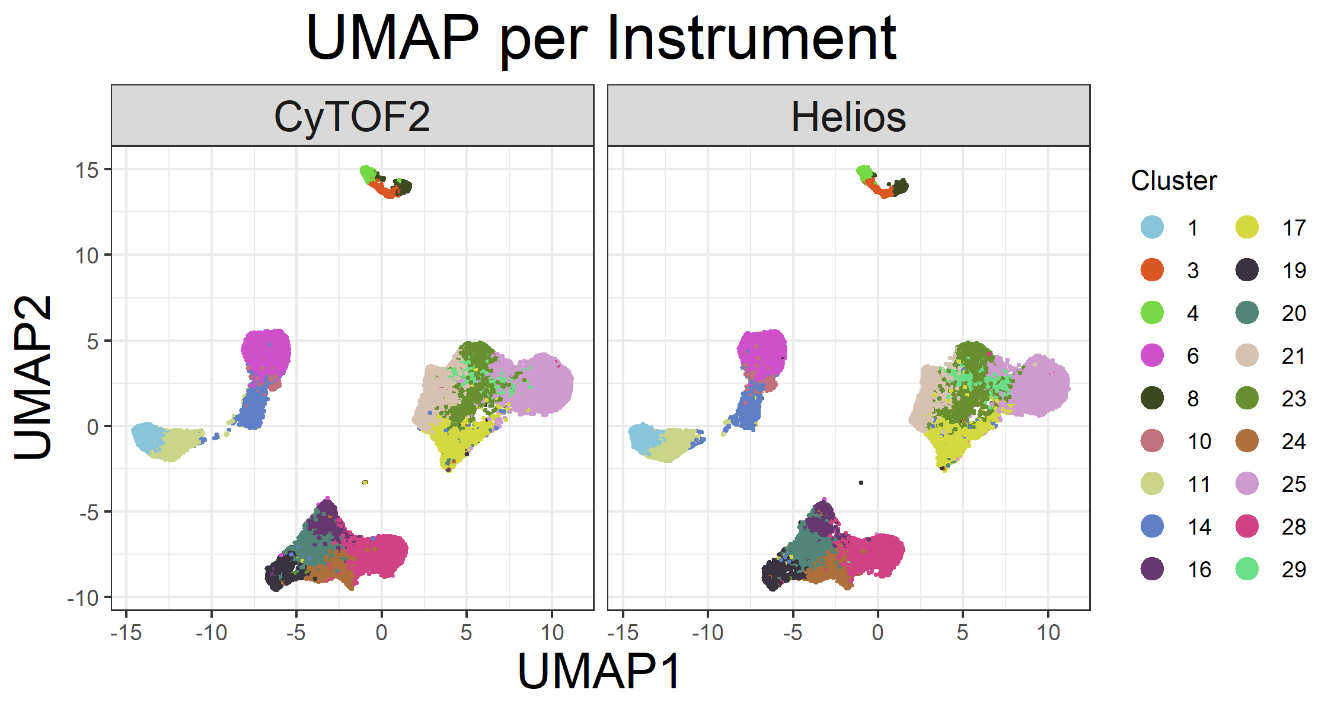


Figure S8. UMAP showing the FlowSOM clusters from the unstimulated data analysis, partitioned by instrument in which the samples were processed in. The colors are mapped to the 18 FlowSOM produced clusters.
